# A pH/ROS cascade-responsive and self-accelerating drug release nanosystem for the targeted treatment of multi-drug-resistant colon cancer

**DOI:** 10.1080/10717544.2020.1797238

**Published:** 2020-07-24

**Authors:** Na Chang, Yufei Zhao, Ning Ge, Liting Qian

**Affiliations:** Department of Radiation Oncology, Anhui Provincial Cancer Hospital (The First Affiliated Hospital of USTC, Division of Life Sciences and Medicine, University of Science and Technology of China), Hefei, China

**Keywords:** ROS amplification, self-accelerate drug release, multidrug resistance

## Abstract

The efficacy of chemotherapeutic agents for colon cancer treatment is limited by multidrug resistance (MDR) and insufficient intracellular release of the administered nanomedicine. To overcome these limitations, we constructed a pH/ROS cascade-responsive and self-accelerating drug release nanoparticle system (PLP-NPs) for the treatment of multidrug-resistant colon cancer. The PLP-NPs comprised a reactive oxygen species (ROS)-sensitive polymeric paclitaxel (PTX) prodrug (DEX-TK-PTX), a pH-sensitive poly(l-histidine) (PHis), and beta-lapachone (Lapa), a ROS-generating agent. We found that PLP-NPs could accumulate in tumor tissue through enhancement of the permeability and retention (EPR) effect, and were subsequently internalized by cancer cells via the endocytic pathway. Within the acidic endo-lysosomal environment, PHis protonation facilitated the escape of the PLP-NPs from the lysosome and release of Lapa. The released Lapa generated a large amount of ROS, consumed ATP, and downregulated P-glycoprotein (P-gp) production through the activity of NQO1, an enzyme that is specifically overexpressed in tumor cells. In addition, the generated ROS promoted the release of PTX from DEX-TK-PTX to kill cancer cells, while ATP depletion inhibited P-gp-mediated MDR. *In vitro* and *in vivo* experiments subsequently confirmed that PLP-NPs induced tumor-specific cytotoxicity and overcame the MDR of colon cancer. Our findings indicate that the use of the PLP-NPs system represents a promising strategy to counter MDR in the treatment of colon cancer.

## Introduction

Colorectal cancer (CRC) is a serious public health challenge and the fourth leading cause of cancer-related deaths globally (Huyghe et al., 2019). It is estimated that, by 2030, the global CRC burden will increase by 60%, representing over 2.2 million new cases and 1.1 million cancer-related deaths (Arnold et al., 2017). Moreover, the incidence rates of CRC are increasing, which is thought to be primarily related to high dietary fat consumption and low dietary fiber intake (Kang et al., 2017). The lifetime risk of developing CRC currently stands at 6%; however, this number is fourfold higher for people with a family history of CRC (Arnold et al., 2017). Although chemotherapy is one of the main strategies for the treatment of CRC, multidrug resistance (MDR) can strongly limit the efficacy of chemotherapy, leading to treatment failure and tumor recurrence in 90% of patients (Robey et al., 2018). The mechanisms underlying MDR are highly complex, and may be influenced by a wide range of factors. The primary mechanism involved in MDR is thought to involve the overexpression of drug efflux pumps related to the ATP-binding cassette superfamily (ABC), such as P-gp, which can rapidly pump multiple chemotherapeutic agents out of the cell, thereby reducing intracellular drug concentrations (Li et al., 2016). Consequently, overcoming P-gp-mediated MDR requires the administration of extremely high drug doses, which may result in severe adverse effects.

Nanomedicines are increasingly being developed to overcome MDR as they can enter cancer cells *via* endocytosis, thus bypassing ABC transporters and ultimately leading to increased intracellular concentrations of cancer-killing drugs (Yan et al., 2010). Nevertheless, there is still a risk of drug efflux by ABC transporters once the encapsulated drug is released into the cytoplasm (Che et al., 2019), and MDR may persist. An effective strategy to effectively overcome MDR might be to combine nanomedicines with inhibition of ABC transporters. For example, as ABC transporters require ATP to function, inhibiting ATP generation may result in the suppression of MDR (Wang et al., 2018).

Insufficient release of nanomedicines in cells can also lead to the failure to attain effective intracellular therapeutic concentrations of a drug (Tang et al., 2017; Wang et al., 2018). Hence, controlled drug release at target sites in response to intracellular stimuli such as ROS, enzyme activity, pH, or glutathione (GSH) may be required to achieve sufficient drug levels and overcome MDR (Zhu et al., 2017; Raza et al., 2019). Consequently, enzyme-responsive nanomedicines have received increasing attention, particularly those that involve NAD(P)H quinone dehydrogenase 1 (NQO1), an enzyme that specifically reduces quinones to hydroquinone through catalytic two-electron reduction (Vasiliou et al., 2006). NQO1 is overexpressed in numerous solid tumors, such as breast, colon, and lung cancers, by up to 100-fold (Ye et al., 2017). Beta-lapachone (Lapa), a substrate of NQO1, is extracted from the bark of the lapacho tree (Siegel et al., 2012; Wang et al., 2019). Lapa is reduced to an unstable hydroquinone through the activity of NQO1, and is rapidly oxidized back to the parent quinone in the presence of molecular oxygen (Siegel et al., 2012; Wang et al., 2019). This redox cycling can generate high concentrations of ROS, and, because the NQO1 enzyme is not involved in the oxidation reaction, ROS is continuously generated to trigger drug release (Li et al., 2019). This suggests that co-delivery of Lapa with a ROS-responsive nanomedicine may specifically increase the level of ROS stimulus in cancer cells and surround tumor tissue with a heterogeneous distribution of ROS. This would lead to a cascade amplification, which would accelerate the release of the drug and eventually overcome the insufficient levels of intracellular drug release. Moreover, Lapa-induced ROS production is accompanied by NAD(P)H/ATP consumption, which can affect the activity of P-gp-associated factors such as HIF-1α, NF-κB, and caspase, ultimately leading to the downregulation of P-gp (Choi et al., 2003; Woo et al., 2006). Therefore, Lapa can effectively reverse MDR through ATP consumption and modulation of P-gp.

Notably, nanomedicines can enter cancer cells *via* the endocytic pathway and are usually trafficked to lysosomes. This process can induce drug degradation or the transfer of the nanomedicines out of the cells, which reduces therapeutic efficacy (Lee et al., 2013). Thus, endosomal sequestration remains a major barrier to effective drug delivery. In addition, to effectively achieve Lapa-mediated amplification of ROS generation, ATP depletion, and P-gp downregulation, Lapa should be released before a chemotherapeutic drug can undergo NQO1-catalyzed redox cycling (Duan et al., 2013).

**Figure 1.**
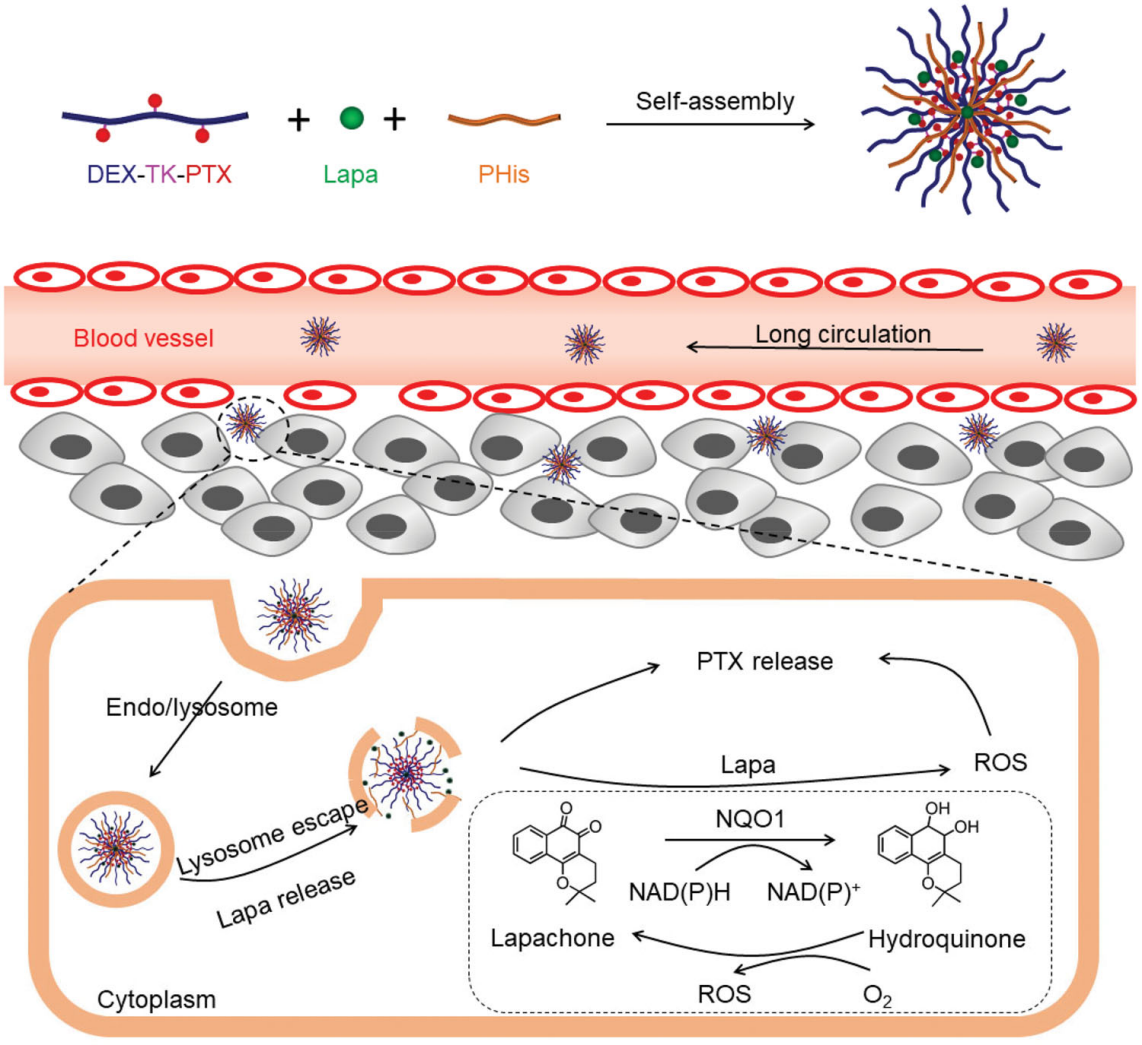
Illustration of the acidic-triggered lysosome escape PLP-NPs with self-amplifiable drug release for MDR CRC treatment.

To solve these problems, we designed and synthesized a pH- and ROS cascade-responsive drug delivery system to overcome MDR in CRC. As illustrated in Figure 1, a ROS-responsive paclitaxel (PTX) polymeric prodrug (DEX-TK-PTX) was synthesized by conjugating PTX to dextran (DEX) through a ROS-cleavable linkage (thioketal, TK). Then, poly(l-histidine) (PHis) and Lapa were encapsulated in DEX-TK-PTX-formed micelles to obtain the final nanosystem, PLP-NPs. Because of excellent biocompatibility of DEX, PLP-NPs may exhibit a long blood-circulation time and accumulate in tumor tissue due to an enhanced permeability and retention (EPR) effect. PLP-NPs would enter into cancer cells *via* the endocytic pathway and become trapped in lysosomes. Under the acidic conditions of lysosomes, PHis would protonate, allowing the PLP-NPs to escape. Simultaneously, Lapa would release and selectively increase the intracellular ROS level in cancer cells through the NQO1-mediateed redox cycle. This facilitates the release of PTX and blockade of ATP-dependent drug efflux, thus increasing the therapeutic effect against MDR colon cancer.

## Experimental

### Thioketal (TK) synthesis

Anhydrous 3-mercaptopropionic acid (3.0 g) was mixed with 3.4 g of acetone, stirred, and then saturated with dry hydrogen chloride at room temperature. After 6 h, the reaction was stopped and the mixture was followed to crystallize under an ice-salt mixture for 24 h. The crude products were then filtered and washed three times each with hexane and cold water. The crude products were then dried in a vacuum desiccator to yield the final product which was then characterized by proton nuclear magnetic resonance (^1^H NMR) and mass spectrometry (MS).

### TK-PTX synthesis

Briefly, a mixture of TK (2 mmol), 1-ethyl-3-(3-dimethylaminopropyl) carbodiimide (EDC; 2.4 mmol), and 4-dimethylaminopyridine (DMAP; 2.4 mmol) were dissolved in DMSO (10 mL) and stirred under N_2_ at 40 °C. After 2 h, 1 mmol of PTX in DMSO was added and the mixture was stirred under N_2_ at 40 °C for 24 h. The mixture was then precipitated with 0.1 M pre-cooled diluted hydrochloric acid and dried under vacuum to obtain a crude product. Then, the crude product was dissolved in methanol and dialyzed against methanol for 24 h to remove unreacted TK and catalysts (the molecular weight cutoff [MWCO] was 500 Da). The final product, TK-PTX, was obtained by drying the mixture under vacuum.

### DEX-TK-PTX synthesis

To synthesize DEX-TK-PTX, TK-PTX (2.4 mmol), EDC (2.8 mmol), and DMAP (2.8 mmol) were dissolved in DMSO (50 mL) and stirred under N_2_ at 40 °C for 0.5 h. Then, DEX (0.1 mmol) was added and reacted under N_2_ at 40 °C for 24 h. Next, the products were purified by dialyzing (MWCO: 5000 Da) against DMSO to remove unreacted small molecules, and then against water to remove DMSO. The products were subsequently lyophilized to obtain the final product. A series of DEX-TK-PTX polymers containing different proportions of PTX were synthesized using the same method and the final amount of PTX in each polymer was measured by UV-vis spectroscopy (UV-2450, SHIMADZU, Japan). The drug binding efficiency (DBE), drug binding content (DBC), and drug binding rate (DBR) were calculated using the formulae given in Equations (1)–(3), respectively, as previously described (Li et al., 2017).

(1) $$\mathrm{DBE}\;(\mathrm{wt}.\%)\;=\;\frac{\text{Mass}\;\text{of}\;\text{PTX}\;\text{in}\;\text{DEX}-\text{TK}-\text{PTX}}{\text{Total}\;\text{mass}\;\text{of}\;\text{feeding}\;\text{drug}}\;\times \;100\%$$

(2) $$\mathrm{DBC}\;(\mathrm{wt}.\%)\;=\;\frac{\text{Mass}\;\text{of}\;\text{PTX}\;\text{in}\;\text{DEX}-\text{TK}-\text{PTX}}{\text{Mass}\;\text{of}\;\text{DEX}-\text{TK}-\text{PTX}}\;\times \;100\%$$

(3) $$\mathrm{DBR}\;=\;\frac{\text{Molar}\;\text{amount}\;\text{of}\;\text{PTX}\;\text{in}\;\text{DEX}-\text{TK}-\text{PTX}}{\text{Molar}\;\text{amount}\;\text{of}\;\text{glucose}\;\text{unit}\;\text{in}\;\text{DEX}-\text{TK}-\text{PTX}}\;\times \;100\%$$

### Determination of the critical micelle concentration (CMC) of DEX-TK-PTX

DEX-TK-PTX was dissolved in PBS in a range of concentrations. Subsequently, Nile Red in DMSO was added to the polymeric solution at a final concentration of 6 × 1 0 ^−7 ^M. A fluorescence spectrometer (RF-5301PC, SHIMADZU, Japan) was then used to determine the fluorescence intensity of each solution (excitation: 557 nm; emission: 601 nm). The CMC value of DEX-TK-PTX was then determined by extrapolating the linear fluorescence intensity at both high and low concentrations.

### Preparation of NPs

The NPs comprising DEX-TK-PTX, Lapa, and Phis were denoted as PLP-NPs, while the control nanoparticles comprising only DEX-TK-PTX and Phis were denoted as PP-NPs. Both NP types were prepared by a nanoprecipitation method. Briefly, DEX-TK-PTX (6 mg), Lapa (1 mg), and PHis (3 mg) were dissolved in DMSO (100 μL) and stirred at room temperature for 2 h. Then, the mixture was slowly added into PBS (pH 7.4, 10 mL) under vigorous stirring. After stirring for 2 h, the solutions were dialyzed (MWCO: 5000 Da) against PBS (pH 7.4) for 6 h at 4 °C, and then filtered through a 0.45-μm Millipore filter to yield the final PLP-NPs. The amounts of PTX and Lapa in the NPs were determined by UV-vis spectroscopy. The drug loading (DL) and entrapment efficiency (EE) were determined using Equations (5) and (6), respectively.

(5) $$\mathrm{DL}\;(\%)\;=\;\frac{\text{mass}\;\text{of}\;\text{drug}}{\text{mass}\;\text{of}\;\text{NPs}}\;\times \;100\%$$

(6) $$\mathrm{EE}\;(\%)\;=\;\frac{\text{the}\;\text{amount}\;\text{of}\;\text{Lapa}\;\text{in}\;\text{NPs}}{\text{total}\;\text{Lapa}\;\text{for}\;\text{preparation}}\;\times \;1000‰$$

To guarantee that the PLP-NPs could rapidly release Lapa at pH 5.5, a series of NPs with different mass ratios of DEX-TK-PTX and Phis (20/1, 10/1, 5/1, 2/1, 1/1, 1/2, 1/5, and 1/10) were prepared using the same method. These were named NP1, NP2, NP3, NP4, NP5, NP6, NP7, and NP8. These NPs were subsequently incubated at pH 7.4, 6.5, or 5.5 for 8 h. Changes in the size of these different NPs were determined by dynamic light scattering (DLS). Then, the NPs were transferred to ultrafiltration centrifuge tubes and centrifuged (5000 rpm, 20 °C, 5 min) to collect the released Lapa. The amount of released Lapa was determined by reversed-phase high-performance liquid chromatography (RP-HPLC). The size change ratio (SCR) and Lapa release level (LRL) were calculated using Equations (7) and (8), respectively.

(7) $$\mathrm{SCR}\;(\%)\;=\;\frac{\text{size of NPs at }0\text{ h }-\text{ size of NPs at }8\;h}{\text{size of NPs at }0\;h}\;\times \;100\%$$

(8) $$\mathrm{LRL}\;(\%)\;=\;\frac{\text{Released mass of Lapa}}{\text{Total mass of Lapa in NPs}}\times \;100\%$$

To track intracellular distribution, coumarin-6 labeled NPs containing DEX-TK-PTX, PHis, and coumarin-6 were also prepared using the same method and defined as PCP-NPs.

### pH and ROS sensitivity analysis

#### pH-responsive NP disassembly assay

Briefly, PLP-NPs were cultured in PBS at pH 5.5 or 7.4 for 12 h, and the average size of the PLP-NPs was then measured by DLS and transmission electron microscopy (TEM).

#### Drug release assay

To investigate the capacity for Lapa release from PLP-NPs under different environments, 2 mL solutions of PLP-NPs (5 mg/mL) were transferred into a dialysis bag (MWCO: 5000 Da) and immersed to PBS at different pHs (pH 5.5, 7.4, and 6.5) and incubated at 37 °C. Aliquots of the dialysates (200 μL) were removed at 0.5, 1, 2, 4, 8, 12, 24, 36, and 48 h, lyophilized, and re-dissolved in methanol. The Lapa concentration was then determined by RP-HPLC. An equal volume of fresh media was added after each aliquot was taken.

To further evaluate the release profiles of PTX, solutions of PLP-NPs in dialysis bags were immersed in PBS (pH 7.4) containing 0.1 or 10 mM of H_2_O_2_ at 37 °C. Aliquots of dialysates were collected as described for the drug release assay. The amount of PTX released was determined by RP-HPLC.

### Intracellular ROS generation

Lapa-induced ROS production was determined with a ROS Assay Kit. Typically, HCT-8/PTX cells seeded onto 60-mm dishes or confocal microscopy dishes were cultured with Lapa, PTX, PP-NPs, or PLP-NPs with or without dicoumarol (60 μM), at a concentration equal to that of Lapa (2 μg/mL). The cells were then incubated with the constituents of the ROS Assay Kit for 30 min. Next, the cells were seeded onto confocal microscopy dishes and then washed, fixed in 4% polyoxymethylene, stained with DAPI, and then imaged by confocal laser scanning microscopy (CLSM). PBS was used as a negative control. In addition, cells seeded on a 60-mm dish were washed, collected by centrifugation, and then quantitatively analyzed by FCM. The ROS-generating ability of NQO1-negative NIH-3T3 cells was also investigated by FCM, as described above.

### *In vitro* cytotoxicity

The cytotoxicity of the PLP-NPs against HCT-8/PTX and HCT-8 cells was determined by MTT assay. Cells were first seeded on 96-well plates (5 × 10^3^ cells per well) and treated with PTX, Lapa, PTX + Lapa, PP-NPs, or PLP-NPs, with or without dicoumarol, for 48 h. The molar ratio of PTX and Lapa was fixed at 1:1. Then, 20 µL of MTT (5 mg/mL) was added into each well and incubated for a further 4 h. The medium was then replaced with 200 µL of DMSO to dissolve the crystals. The absorbance of formazan was determined using a Microplate reader. IC_50_ values were calculated using GraphPad Software (Prism 8).

We also investigated the ability of PLP-NPs to inhibit the growth of NIH-3T3 cells. Cells (1 × 10^3^ cells per well) were seeded onto 96-well plates and cultured with PP-NPs or PLP-NPs for 48 h. Cell viability was evaluated using the MTT method, as described above.

### *In vivo* antitumor activities

HCT-8/PTX tumor-bearing mice were randomly divided into six groups (PBS, PTX, Lapa, PTX + Lapa, PP-NPs, and PLP-NPs) after tumor volume had reached approximately 50–100 mm^3^. Each group consisted of six mice. The mice were intravenously injected with different drug formulations at a PTX equivalent dose of 5 mg/kg on days 0, 3, and 6. The body weight and tumor volume of each mouse were measured every 2 days. The equation: volume = L × S^2^/2 was used to calculate tumor volume, where L and S represent the longest and shortest tumor dimensions, respectively. The mice were sacrificed on day 20. The tumor and major organs (liver, heart, spleen, kidneys, and lung) of each mouse were harvested and weighed, and then stained with hematoxylin and eosin (H&E) for histological analysis.

### Statistical analysis

All data are presented as means ± standard deviation (SD). Differences between two groups were analyzed by the Student’s *t*-test. One-way analysis of variance (ANOVA) was used to compare means between multiple groups. A *p*-value <.05 was considered significant.

## Results and discussion

### DEX-TK-PTX characterization

Synthesizing polysaccharide prodrugs through the conjugation of chemotherapeutic drugs to polysaccharide backbones has developed into an excellent strategy with which to improve drug bioavailability and reduce systemic toxicity. DEX, a glucose homopolysaccharide composed of D-glucose units linked by α-(1-4) glycosidic bonds, exhibits excellent biocompatibility and is widely used to develop polysaccharide prodrugs (Curcio et al., 2020). Compared with polyethylene glycol (PEG), using DEX as a drug carrier resulted in reduced nonspecific protein adsorption and higher stability in plasma (Manchun et al., 2015). Because of its excellent biocompatibility, in this study, we employed DEX as the polymer material with which to deliver chemotherapeutic drugs.

**Figure 2.**
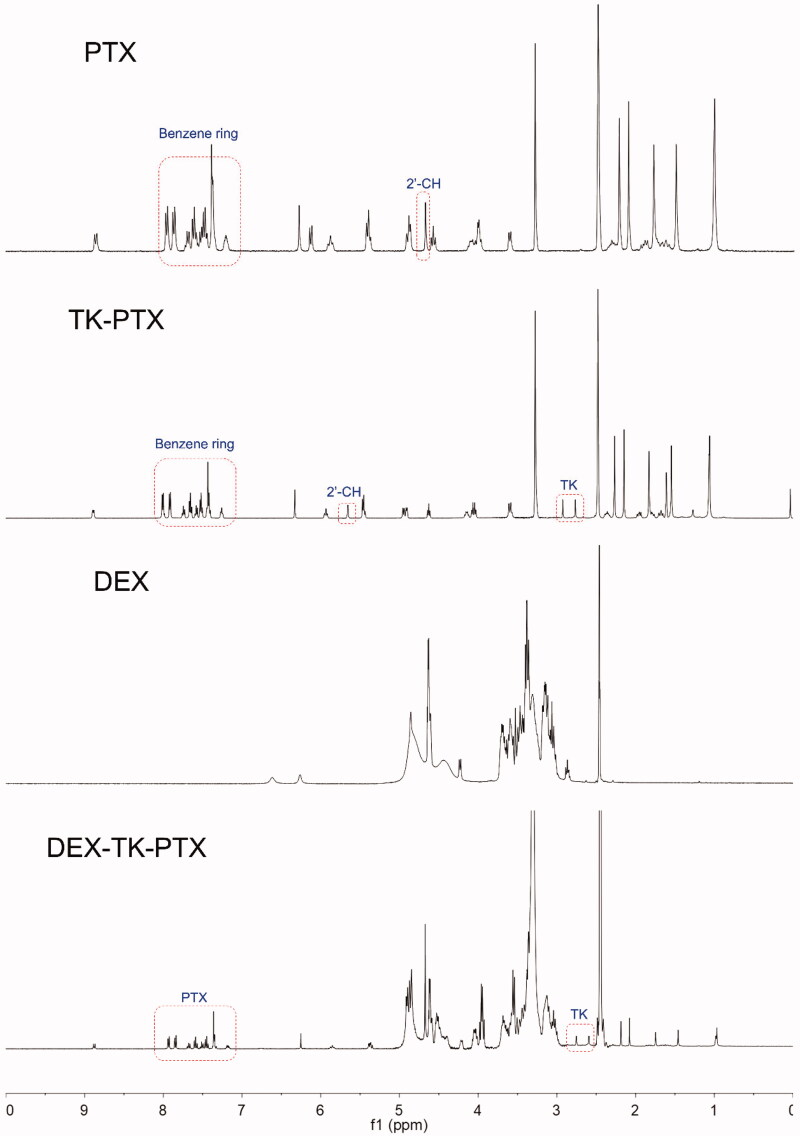
^1^H NMR spectrum of PTX, TK-PTX, DEX, and DEX-TK-PTX in DMSO-d6.

We first synthesized the PTX prodrug, DEX-TK-PTX, and the synthetic route is presented in Supplementary Figure S1. The ROS-sensitive linkage was prepared first and characterized by ^1^H NMR and MS (Supplementary Figure S2). The results demonstrated that TK had been successfully synthesized, which was consistent with a previous report (Chen et al., 2016; Xu et al., 2020). Next, TK-PTX was prepared through an esterification reaction, and was also characterized by ^1^H NMR and MS (Figure 2 and Supplementary Figure S3). The ^1^H NMR spectrum showed peaks at 2.8–3.2 ppm, which represented the methylene in TK, while the peaks at 6.9–7.5 ppm were attributed to the benzene signals of PTX (Figure 2). We also found that the 2′-CH peak derived from conjugated PTX shifted from 4.8 ppm to 5.6 ppm when compared with free PTX. The MS spectrum showed a peak at 1088.3 that corresponded to [M-H]^−^, which was consistent with the calculated value (Supplementary Figure S3). These results demonstrated that TK-PTX had been successfully prepared. Finally, TK-PTX was conjugated to DEX to create the final product, DEX-TK-PTX, the structure of which was also confirmed by ^1^H NMR (Figure 2). Peaks at 7.2–7.9 ppm represented the benzene rings of PTX, the signals at 2.9–3.7 and 4.3–5.0 ppm were assigned to the glycoside protons of DEX, and the characteristic peaks of TK were evident at 2.6–2.7 ppm. Collectively, these results indicated that DEX-TK-PTX had been successfully prepared.

Our design included incorporating Lapa, a ROS producer and P-gp inhibitor, into the core of the DEX-TK-PTX NPs. Therefore, to avoid the premature release of Lapa into the blood, DEX-TK-PTX required a low CMC to withstand the hemodilution effect. Furthermore, a high drug loading efficacy was also required for effective drug delivery. To obtain an optimal PTX prodrug, we first tested a series of DEX-TK-PTX conjugates. The DBEs, DBCs, and DBEs of these PTX prodrugs were assessed by UV-vis spectrophotometry and their CMC values were determined using Nile red as a fluorescence probe. These data are presented in Supplementary Table S1. The results showed that the CMC for DEX-TK-PTX was lowest (4.4 μg/mL) when the DBR of PTX in the prodrug was 20.2%. Consequently, this PTX prodrug was employed for all subsequent experiments.

### Preparation and characterization of PLP-NPs

We employed PHis to counter the barrier represented by the lysosomes, so as to release Lapa more rapidly and activate Lapa-induced ROS generation and ATP depletion. PHis contains an imidazole group that will become protonated under weakly acidic conditions, resulting in hydrophobic/hydrophilic transformation, the disassembly of PLP-NPs, and the release of Lapa (Jiang et al., 2018). The protonation process requires an abundance of H^+^ ions, which leads to lysosome and endosome membrane disruption through the proton-sponge effect and results in the accelerated release of PLP-NPs from endo-lysosomes (Herranz‐Blanco et al., 2016). Acid–base titration was used to evaluate the buffering capacity of PHis. The titration curve of PHis is depicted in Supplementary Figure S4, and shows that the control group (NaCl) had no buffering capacity between pH 5.5 and 7.4. In contrast, PHis exhibited a marked buffering capacity in the same pH range (27.5%).

**Figure 3.**
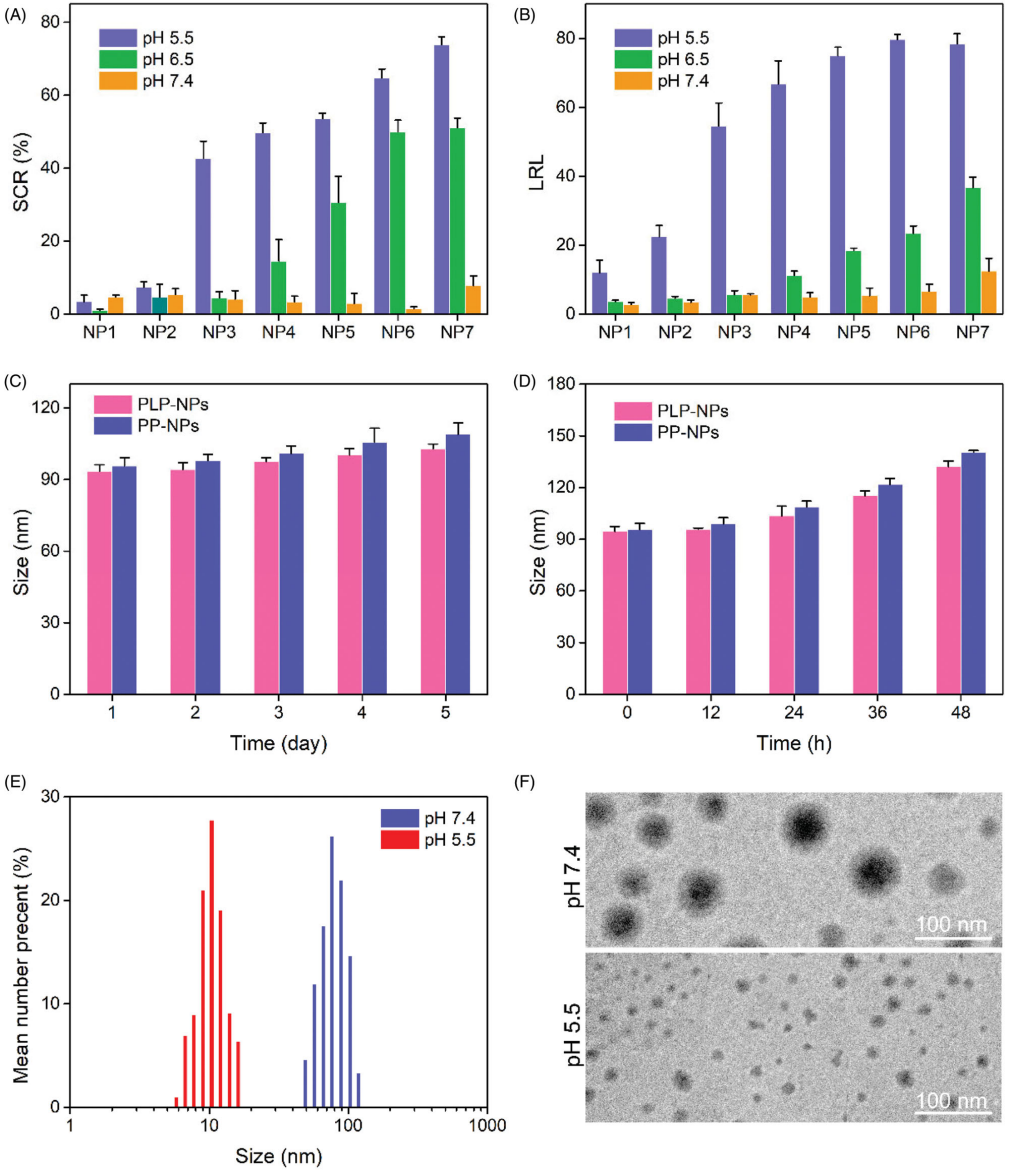
(A, B) Size change ratio (SCR, A) and Lapa release level (LRL, B) of NP1, NP2, NP3, NP4, NP5, NP6, and NP7, after incubation at pH 7.4, 6.5, and 5.5 for 8 h (*n* = 5). (C–D) Particle size of PLP-NPs and PP-NPs in PBS at pH 7.4 (C) and in RPMI-1640 containing 10% serum (D) (*n* = 5). (E–F) DLS results (E) and TEM images (F) of PLP-NPs cultured at pH 7.4 or pH 6.5 for 8 h.

We subsequently prepared the NPs by coprecipitation. To guarantee that PLP-NPs could release Lapa effectively at pH 5.5, we prepared a series of NPs (NP1–8) with different DEX-TK-PTX/PHis mass ratios. We then determined the changes in size as well as the amount of Lapa released by these NPs (SCR and LRL, respectively) after 8 h incubation at pHs equal to those of blood, tumor extracellular environment, and lysosomes (pH 7.4, 6.5, and 5.5, respectively). Because the size of NP8 exceeded 400 nm, we did not determine its exact size. As shown in Figure 3(A,B), none of the NPs showed a significant size change at pH 7.4 (SCR <8%), and the LRL values were lower than 15%. These data indicated that these NPs were stable at blood pH. When the pH value was reduced to 6.5, the sizes of NP1, NP2, and NP3 did not change significantly (SCR <5%), and the LRL was <7%. However, the SCR and LRL of NP4, NP5, NP6, and NP7 were 14.5%/11.2%, 30.5%/18.5%, 50.0%/23.4%, and 51.1%/36.7%, respectively. In addition, there was a marked increase in the SCR and LRL of these NPs at the pH value similar to that observed in lysosomes (pH 5.5). This suggested that these NPs could release Lapa rapidly in the acidic conditions found in the lysosome. Moreover, the SCR and LRL of NP3 at pH 7.4 and 6.5 were lower than those of NP4, NP5, NP6, and NP7, but higher than those of NP1 and NP2 at pH 5.5, suggesting that NP3 would remain stable in the conditions found in the blood and the cancer extracellular environment, but could rapidly release Lapa in the acidic environment of the lysosomes. Consequently, the mass ratio of DEX-TK-PTX and PHis was fixed at 5:1 for all subsequent experiments.

Further details relating to the PLP-NPs and PP-NPs comprising DEX-TK-PTX and PHis at a mass ratio of 5:1 are presented in Table 1. Both nanosystems were approximately 90 nm in size and showed a narrow particle dispersion index (PDI) lower than 0.2. Moreover, both NP systems had a high drug loading content (DLC), which was 15.3% and 16.2% for PTX, respectively, while the PLP-NP DLC for Lapa was approximately 3.2%. To confirm the predicted physiological stability of these NPs, the size changes of the PLP-NPs and PP-NPs were determined by DLS over time in a simulated solution (Figure 3(C,D)). We found that both NPs remained stable in PBS (pH 7.4) over 7 days, or in RPMI 1640 containing 10% serum over 48 h. The high stability of the PLP-NPs and PP-NPs was attributed to the nonspecific protein adsorption of DEX and the low CMC value of DEX-TK-PTX. We also employed DLS and TEM to evaluate the pH-responsivity of the PLP-NPs (Figure 3(E,F)). The mean size of the PLP-NPs at pH 7.4 was approximately 90 nm, while TEM further showed that these PLP-NPs adopted a spheroid morphology. However, after culture at pH 5.5 for 12 h, the NPs had completely collapsed, which was consistent with a previous report (Herranz‐Blanco et al., 2016).

**Table 1.** Characterization of NPs (*n* = 5).

NPs	Size (nm)	Zeta (mV)	DL of PTX (%)	DL of Lapa (%)	EE of Lapa (%)
PLP-NPs	94.5 ± 3.0	−9.1 ± 1.8	16.2 ± 2.1	4.1 ± 0.8	93.2 ± 2.1
PP-NPs	95.6 ± 3.5	−9.7 ± 3.2	16.8 ± 1.9	–	–

### pH and ROS-responsive drug release

**Figure 4.**
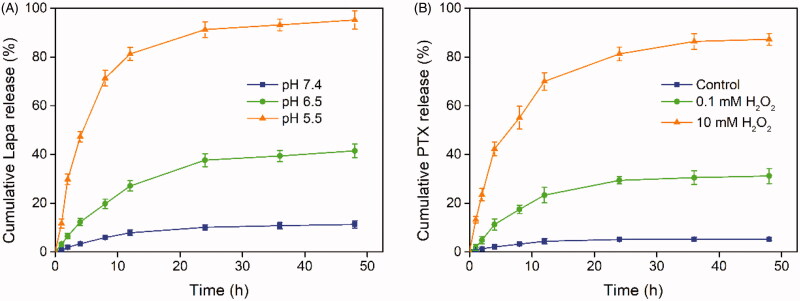
Stimuli-responsive drug release analysis. (A) Lapa release profile of the PLP-NPs at pH 7.4, 6.5, and 5.5, respectively (*n* = 5). (B) Cumulative release of PTX from PLP-NPs in the presence of 0, 0.1, and 10 mM H_2_O_2_ (*n* = 5).

We hypothesized that PLP-NPs could rapidly disassemble in lysosomes to release Lapa and that the released Lapa could generate large amounts of ROS to trigger the release of PTX. We also hypothesized that spatial and temporal disparities in drug release would significantly accelerate drug release and overcome drug resistance. To confirm this hypothesis, we performed a drug release assay using a dialysis method. To analyze Lapa release, release media at pH 7.4, 6.5, or 5.5 was used to simulate the pH values of blood, tumor tissue, and lysosomes, respectively. The relative amounts of Lapa released were then determined by RP-HPLC (Figure 4(A)). Lapa was released slowly, and in low amounts, at both pH 7.4 and 6.5. After 48 h of incubation, only 11.3% and 41.5% of Lapa were released from PLP-NPs at pH 6.5 and pH 7.4, respectively. In contrast, when the pH value was reduced to 5.5, the release rate of Lapa exhibited a marked increase, with 95.2% of Lapa having been released after 48 h of culture. The pH-dependent capacity for Lapa release significantly enhanced the pH-responsive disassembly of PLP-NPs.

Next, we investigated the ROS-responsive PTX release behavior of PLP-NPs using H_2_O_2_ as a trigger. As shown in Figure 4(B), PTX exhibited a slow release rate in the absence or presence of minute quantities of H_2_O_2_ over a 48 h period (5.3% and 31.2% of PTX, respectively). However, in the presence of 10 mM H_2_O_2_, the amount of PTX released reached 87.3%, which may have been related to cleavage of the TK linker in the presence of high ROS concentrations. These results consistently demonstrated that the PLP-NPs remained stable in a physiological environment and successfully achieved pH and ROS dual-responsive drug release, thereby reducing the risk of potential side effects.

### The promotion of endo-lysosomal escape

**Figure 5.**
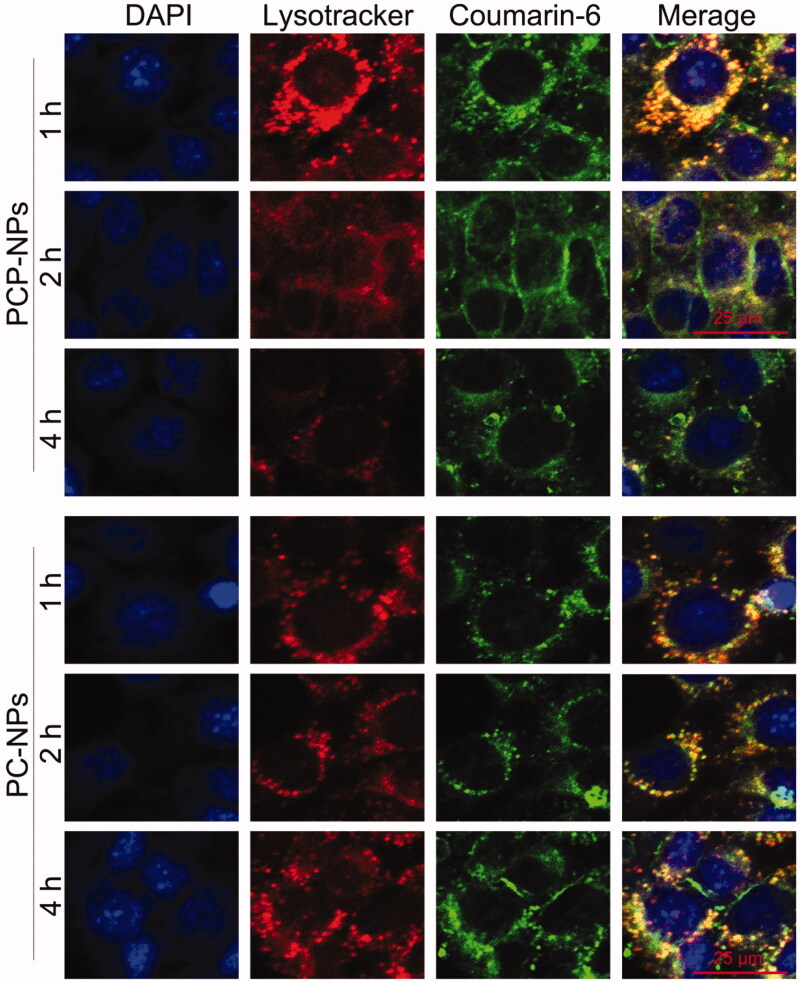
CLSM images of PHis-mediated lysosomal escape in HCT-8/PTX cells treated with coumarin-6-loaded PCP-NPs (with PHis) or PC-NPs (without PHis) for 1, 2, and 4 h, respectively.

The high molecular weight of DEX can cause it to accumulate in lysosomes, leading to the entrapment of DEX-based NPs within lysosomes (Li et al., 2017). However, the long-term retention of NPs within lysosomes is not conducive to the efficient delivery of drugs into the cytoplasm of tumor cells (Paillard et al., 2010; Lale et al., 2015). To address this, a pH-sensitive polymer, PHis, was incorporated into the PLP-NPs to facilitate the release of PLP-NPs from the lysosomes *via* the proton sponge effect, thus achieving the release of both Lapa and PTX. To confirm this hypothesis, we used CLSM to observe the intracellular trafficking of PLP-NPs in HCT-8/PTX cells. To evaluate the lysosome-escape effect, we incubated the cells with coumarin-6 loaded NPs (PCP-NPs, composed of DEX-TK-PTX, PHis, and coumarin-6) or control NPs (PC-NPs, formed by DEX-TK-PTX and coumarin-6). In the PC-NPs treatment group, an extensive distribution of green fluorescence arising from coumarin-6 colocalized with the red fluorescence emitted by LysoTracker (Figure 5), indicating an abundance of PC-NPs in the lysosomes, even after 4 h of incubation. In contrast, PCP-NPs were able to escape from the lysosomes after only 2 h of incubation, as evidenced by an abundance of green fluorescence in the cytoplasm of cells. This was further supported by the fact that this green fluorescence was not colocalized with the red fluorescence of the LysoTracker. This result confirmed that PLP-NPs can rapidly and effectively escape from lysosomes in cancer cells.

### Lapa-induced intracellular ROS production, acceleration of PTX release, and tumor-specific cytotoxicity

**Figure 6.**
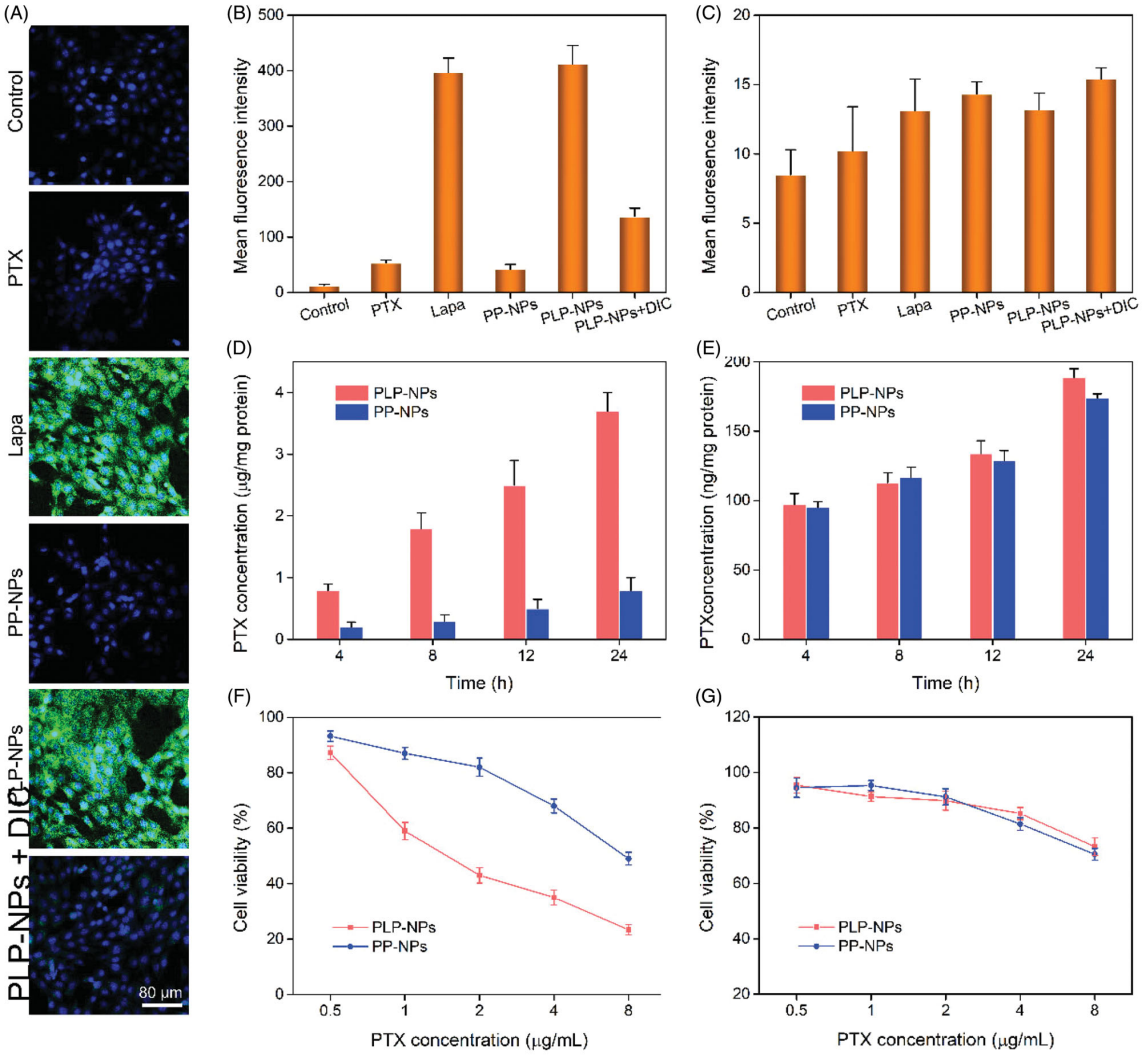
Lapa-mediated ROS production assay. (A) CLSM images of HCT-8/PTX cells treated with PTX, Lapa, PP-NPs, PLP-NPs, or PLP-NPs + DIC, and then stained with DCFH-DA (green fluorescence) and DAPI (blue fluorescence). (B, C) FCM detection of intracellular DCF intensity in cancer HCT-8/PTX cells (B) and normal NIH-3T3 (C) cells after treatment with PTX, Lapa, PP-NPs, PLP-NPs, or PLP-NPs + DIC (*n* = 5). (D, E) The amount of PTX released intracellularly in HCT-8/PTX (D) and NIH-3T3 cells (E) after treatment with PLP-NPs or PP-NPs for 4, 8, 12, or 24 h (*n* = 5). (F, G) The cytotoxicity of PLP-NPs or PP-NPs in HCT-8 cancer cells (F) and in normal NIH-3T3 cells (G) (*n* = 6).

The ability of Lapa to induce ROS generation was investigated in HCT-8/PTX cells, which overexpress the enzyme NQO1 (Vieira et al., 2015; Liu et al., 2020). We used 2′,7′-dichlorofluorescin diacetate (DCFH-DA) as a probe to stain intracellular ROS. DCFH-DA has no fluorescence signal, but can be quickly oxidized into dichlorofluorescein (DCF), which emits strong green fluorescence. HCT-8/PTX cells were treated with Lapa, PTX, PP-NPs, PLP-NPs, or PLP-NPs plus dicoumarol (DIC) for 2 h and then stained with DCFH-DA. The relative amounts of ROS generated were then measured by CLSM and FCM. As shown in Figure 6(A), there was almost no fluorescence signal in cells incubated with PTX and PP-NPs. In contrast, cells incubated with Lapa and PLP-NPs exhibited strong green fluorescence, indicative of high levels of intracellular ROS. However, when PLP-NPs were combined with DIC, a NQO1 inhibitor, the fluorescence signal was significantly lower than with PLP-NPs alone, suggesting that Lapa-induced ROS production was dependent on NQO1. We further evaluated the ability of Lapa to generate ROS by FCM, and obtained results like those of CLSM (Figure 6(B)). The intracellular levels of ROS in cells treated with either Lapa- or PLP-NPs were 13.2- and 14.1-fold higher than those of the control group, respectively. Moreover, to demonstrate the tumor-specific production of ROS, we used a normal cell line (NIH-3T3 cells with low NQO1 expression) as a control. As shown in Figure 6(C), all drug-treated groups showed negligible levels of fluorescence, with no significant differences between groups. These data indicated that Lapa-mediated ROS generation was dependent on the NQO1 enzyme and was thus tumor-specific.

We hypothesized that Lapa-induced intracellular ROS generation could promote the release of PTX from PLP-NPs. To confirm this, we measured the intracellular active PTX level in HCT-8/PTX cells by HPLC after treatment with PLP-NPs and PP-NPs. As presented in Figure 6(D), the intracellular levels of prototype-PTX increased with increasing incubation time. At 4, 8, 12 or 24 h of incubation, the intracellular levels of active PTX in the PLP-NPs group were 3.8-, 1.3-, 2.5-, and 3.9-fold higher than those of the PP-NPs group, respectively. Moreover, when HCT-8/PTX cells were co-incubated with DIC and PLP-NPs, the intracellular concentration of PTX decreased markedly. Similarly, in NIH-3T3 cells (Figure 6(E)), only minimal amounts of PTX were detected in each group. These results clearly demonstrated that Lapa can rapidly produce an abundance of ROS in cancer cells through the activity of NQO1, resulting in significantly accelerated levels of drug release.

Encouraged by the tumor-specific intracellular drug release of PLP-NPs, we next evaluated tumor-specific cytotoxicity using MTT assays. In normal NIH-3T3 cells, which exhibit low expression levels of NQO1, we found that PP-NPs and PLP-NPs showed only weak levels of cytotoxicity, even when the concentration of PTX reached 5 μg/mL (82.3% and 85.3% of cells incubated with PP-NPs and PLP-NPs survived after 48 h, respectively; Figure 6(F)). In comparison, less than 30.2% of cells survived after treatment with free PTX at a dose of 5 μg/mL. These results suggested that the PLP-NPs did not exert any notable cytotoxicity in normal cells. In contrast, when incubated with HCT-8 cancer cells (Figure 6(G)), PLP-NPs induced high levels of cytotoxicity when compared with incubation with either free PTX or PP-NPs. However, incubation with PP-NPs resulted in the highest cell viability (with a 56.3% survival rate), even at a drug dose of 5 μg/mL. In addition, when we introduced a NQO1 inhibitor, DIC, we found that PLP-NP-induced cytotoxicity was significantly reduced. These results may be explained by the fact that PTX cannot be released from PP-NPs and PLP-NPs in normal cells because the TK linker remains stable under low ROS conditions. However, in cells that overexpress NQO1, the amount of Lapa released was sufficient to generate enough ROS through NQO1-mediated catalysis to facilitate the release of PTX, thereby inducing a cytotoxic effect. Therefore, these results provide good evidence that PLP-NPs can specifically induce cytotoxicity in cancer cells, which minimizes undesired side effects.

### Overcoming MDR *in vitro* and analysis of the associated mechanisms

**Figure 7.**
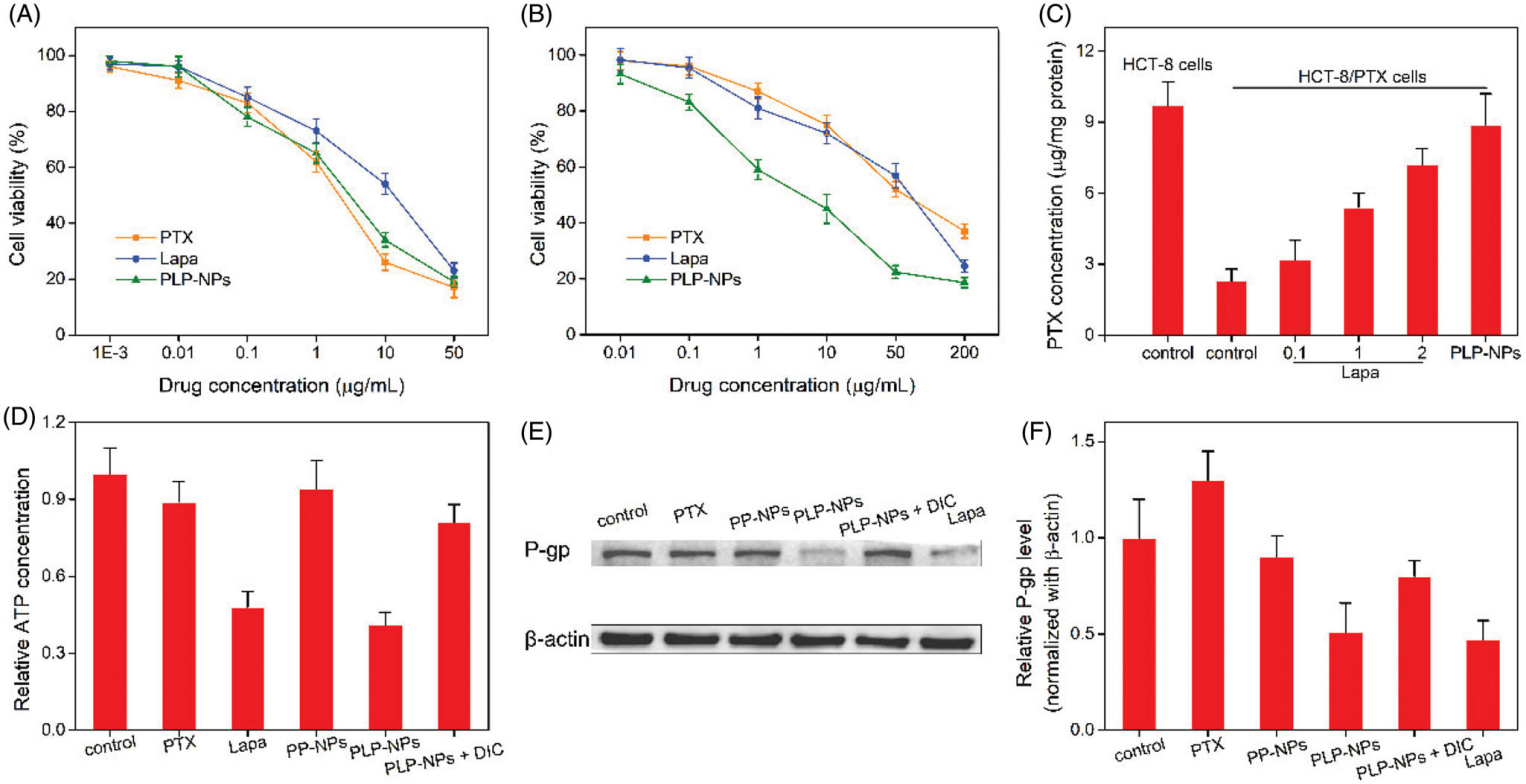
Investigating the ability to overcome MDR *in vitro*. (A, B) Cell viability of HCT-8 cells (A) and HCT-8/PTX cells (B) after incubation with PTX, Lapa, or PLP-NPs (*n* = 6). (C) The amount of PTX in HCT-8 and HCT-8/PTX cells after treatment with different drug formulations (*n* = 5). ‘Control’ represents cells incubated with free PTX. (D) The level of ATP in HCT-8/PTX cells after treatment with PTX, Lapa, PP-NPs, PLP-NPs, or PLP-NPs + DIC (*n* = 5). (E, F) Western blotting images (E) and quantitative analyses (F) of P-gp expression in HCT-8/PTX cells after treatment with PTX, Lapa, PP-NPs, PLP-NPs, or PLP-NPs + DIC (*n* = 5).

To evaluate the ability of PLP-NPs to counter MDR *in vitro*, we used MTT assays to determine the cytotoxicity of PLP-NPs against drug-resistant HCT-8/PTX cells and drug-sensitive HCT-8 cells. In the drug-sensitive cells, all the formulations showed a similar ability to inhibit cell growth (Figure 7(A)). In contrast, in the drug-resistance HCT-8/PTX cells, the free PTX only marginally inhibited cell growth (Figure 7(B)). The half-maximal inhibitory concentration (IC_50_) of PTX was 45.3 μg/mL, which was 43-fold higher than that of PTX in HCT-8 cells, demonstrating that HCT-8/PTX cells were significantly more resistant to PTX. When combined with Lapa (PLP-NPs), PTX cytotoxicity was greatly enhanced, presenting an IC_50_ value of 5.3 μg/mL, which was 38-fold lower than that of free PTX. These data provide convincing evidence that PLP-NPs can effectively overcome MDR *in vitro*.

The capacity of PLP-NPs to reverse MDR *in vitro* may be attributed to Lapa-mediated ATP depletion. NQO1-catalyzed redox cycling of Lapa is reported to significantly increase the levels of intracellular calcium, resulting in mitochondrial membrane depolarization and ATP consumption (Blanco et al., 2010). Moreover, the ATP-dependent and P-gp-induced efflux of chemotherapeutic agents is one of the major biological mechanisms responsible for MDR (Arora & Shukla, 2003). This suggests that Lapa-induced ATP depletion may inhibit P-gp-mediated drug efflux and ultimately overcome P-gp-mediated drug resistance. To confirm this hypothesis, we determined the influence of Lapa on the relative levels of ATP and P-gp in MDR cells. First, we measured the expression levels of P-gp in HCT-8/PTX cells and HCT-8 cancer cells by western blotting. As presented in Supplementary Figure S5, the levels of P-gp protein in HCT-8/PTX cells were 15-fold higher than those in HCT-8 cells, suggesting that the high level of P-gp expression was the key factor responsible for PTX resistance in HCT-8/PTX cells. Next, we used HPLC to determine the accumulation of PTX in drug-resistant cells to ascertain whether Lapa inhibited the P-gp-induced drug efflux effect. As shown in Figure 7(C), the intracellular drug concentration in the free PTX treatment group was relatively low after 12 h of treatment. When Lapa and PTX were combined, the intracellular concentration of PTX increased markedly in a manner that was dependent on the concentration of Lapa. The intracellular concentration of PTX in the presence of 0.1, 1, and 2 μg/mL of Lapa was increased by 1.4-, 2.3-, and 3.2-fold, respectively. Similarly, the intracellular concentration of PTX in the PLP-NPs treatment group was 3.9-fold higher than that of the free PTX treatment group. These results clearly suggest that Lapa suppressed P-gp-mediated drug efflux.

To further confirm the mechanism underlying the ability of PLP-NPs to reverse MDR, we measured the concentration of ATP in drug-resistant cells after treatment with different drug formulations. As illustrated in Figure 7(D), the intracellular concentration of ATP in MDR cells was clearly depleted following treatment with Lapa and PLP-NPs. However, after the introduction of DIC (PLP-NPs + DIC), the intracellular level of ATP increased by approximately 91% in comparison with PLP-NPs treatment alone. These results suggested that Lapa-mediated ROS production was accompanied by ATP consumption. In addition, Lapa-induced ATP depletion is known to affect several P-gp-related factors, including caspase, HIF-1α, and NF-κB, which eventually lead to the downregulation of P-gp expression (Siegel et al., 2012). To further clarify this, we measured the expression of P-gp in HCT-8/PTX cells after incubation with different drug formulations. As presented in Figure 7(E,F), both Lapa and PLP-NPs could significantly suppress the expression of P-gp. However, the inhibitory effect of PLP-NPs on P-gp expression was markedly reduced by the addition of DIC, indicating that Lapa-induced ROS generation also has a role in the regulation of P-gp expression. Collectively, these data indicate that PLP-NPs can effectively overcome P-gp-mediated MDR by reducing the expression of P-gp and by eliminating ATP, thereby greatly enhancing anti-tumor efficacy.

### Pharmaceutics and *in vivo* biodistribution

**Figure 8.**
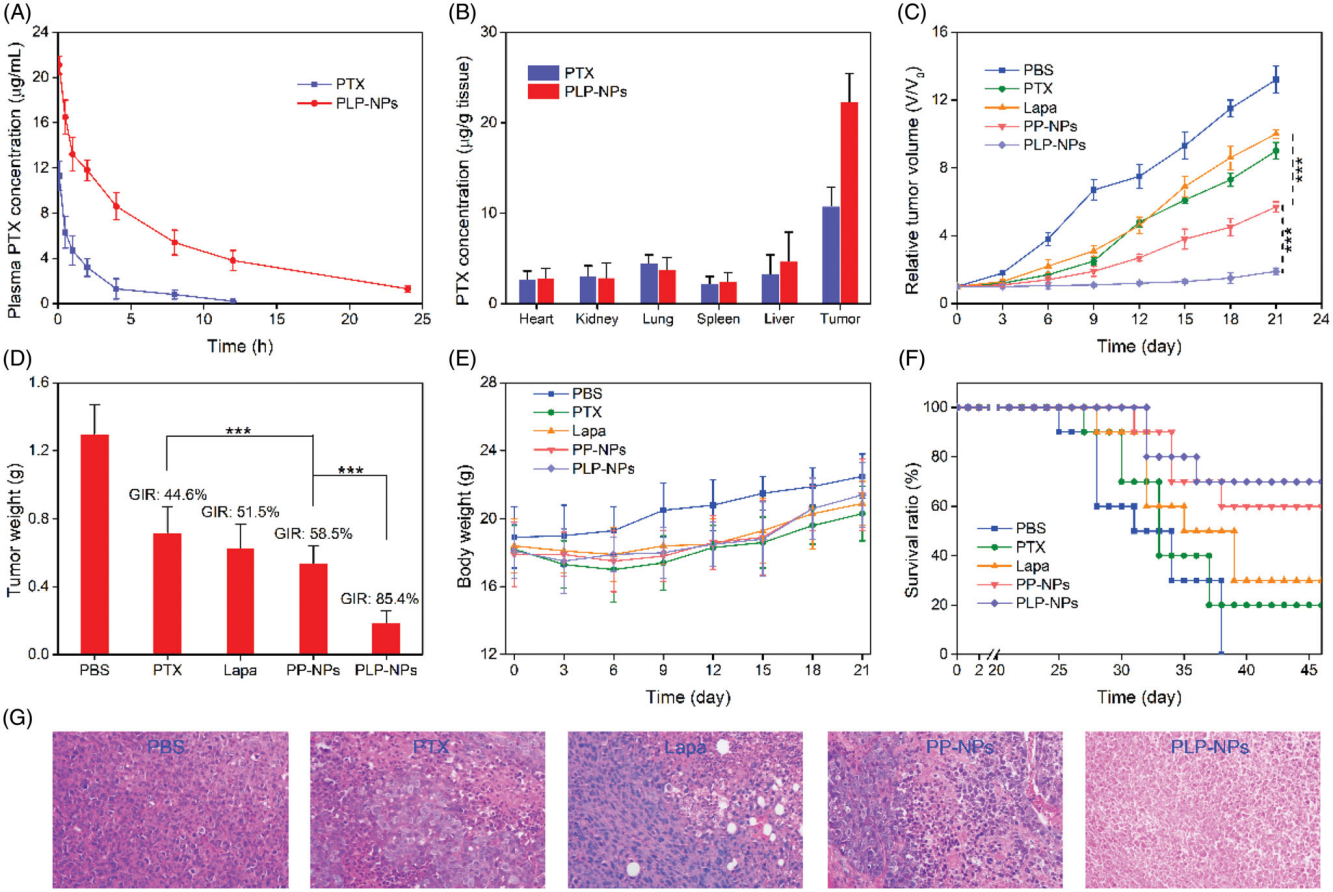
*In vivo* studies. (A) Plasma drug concentration as a function of time with PTX and PLP-NPs administration (*n* = 5). (B) PTX level in the major tissues following the administration of PTX and PLP-NPs for 12 h (*n* = 5). (C) Relative tumor volumes after treatment with different drug formulations (*n* = 6). (D) Tumor weight on day 21 in different drug treatment groups (*n* = 6). (E) The body weights of mice during the treatment period (*n* = 6). (F) Survival ratio of mice after different treatments (*n* = 6). (G) Hematoxylin and eosin (H&E) staining of tumor tissues after different treatments.

It is well established that DEX-based nanomedicine can effectively prolong the blood-circulation times of chemotherapeutic agents, an important characteristic for EPR-mediated passive tumor targeting. To confirm this, we evaluated the *in vivo* pharmacokinetics of PLP-NPs. In this study, we used Sprague–Dawley (SD) rats as an animal model to monitor the blood-circulation time of PLP-NPs *in vivo*. After the single administration of PTX and PLP-NPs, we collected blood samples at pre-established time points and measured plasma PTX concentration by HPLC. As presented in Figure 8(A), when protected by DEX, PLP-NPs had a relatively longer circulation time, exhibiting a half-life 5.3-fold longer than that of free PTX. The area under the concentration curve of PLP-NPs was 3.2-fold higher than that for free PTX. Furthermore, approximately 5.3% of PLP-NPs remained in the blood 10 h after injection compared with only 0.2% for free PTX. Next, we investigated how longer circulation times in the blood might promote the accumulation of PLP-NPs at the tumor site in HCT-8/PTX tumor-bearing mice. As shown in Figure 8(B), 24 h after injection, 5.3% of PLP-NPs had accumulated in tumor tissue, compared with 3.2% of free PTX, indicating that PLP-NPs can deliver drugs effectively to the site of tumors and that this was based on the EPR effect.

### *In vivo* antitumor efficacy

As we found that our nanosystem could overcome MDR *in vitro* and lead to the accumulation of high levels of nanomedicine at tumor sites, we next investigated the efficacy of this treatment *in vivo*. HCT-8/PTX tumor-bearing mice were randomly divided into five groups when the tumor size had reached approximately 80 mm^3^ (*n* = 6). Mice in these groups were then treated with PBS, PTX, Lapa, PP-NPs, or PLP-NPs (using an equivalent dose of 5 mg/kg PTX and 2 mg/kg Lapa) every 3 days for a total of 9 days. As illustrated in Figure 8(C), tumor growth was suppressed by all drug formulations when compared with the PBS group. Importantly, based on the synergistic effect of PTX and Lapa, PLP-NPs exhibited a significantly better treatment efficacy than the other groups. All mice were euthanized on day 21 and the tumors harvested and weighed to assess tumor growth inhibition rates (GIRs) for the different groups over the same time points. Tumor weight was consistent with the volume data (Figure 8(D)). In addition, PLP-NPs exhibited the highest GIRs (88.3%), which were 3.2-, 2.5-, and 3.6-fold higher than those for PTX, Lapa, and PP-NPs, respectively (Figure 8(D)). Importantly, mice in the PLP-NPs group showed no weight loss during the treatment period, whereas mice treated with PTX showed a clear loss of weight (Figure 8(E)). As expected, based on their better antitumor efficacy, PLP-NPs also prolonged the survival time of the animals (Figure 8(F)). In addition, H&E staining (Figure 8(G)) also indicated that cells in the PLP-NPs treatment group shrank significantly, showing distinct chromatin condensation, which indicated that PLP-NPs induced greater levels of necrosis than the other groups. These results demonstrated that the PLP-NPs could effectively overcome MDR *in vivo*. We hypothesize that the mechanism underlying this effect can be explained as follows. First, DEX protection significantly extended the blood-circulation time of the PLP-NPs, resulting in high accumulation at tumor sites *via* the EPR effect (Kinoshita et al., 2017). Following internalization, PHis protonation in the acidic environment of the endosomes and lysosomes led to the escape of PLP-NPs and the release of Lapa (Gao et al., 2019). The released Lapa could then induce ROS generation, which subsequently led to PTX release. Finally, Lapa induced the depletion of ATP and downregulation of P-gp, acting in combination with PTX to reverse MDR.

## Conclusions

In this study, we developed and characterized a pH and enzyme dual-responsive nanomedicine (PLP-NPs) with self-accelerating drug release and the ability to overcome MDR. A combination of *in vitro* and *in vivo* studies demonstrated that PLP-NPs could increase intracellular ROS level, enhance intracellular drug concentrations, and deplete ATP levels in MDR cancer cells. PLP-NPs were also found to downregulate P-gp expression in MDR cancer cells, prolong drug circulation in the blood, enhance the accumulation of drugs in tumor tissue, suppress tumor growth, and extend the survival of mice. In summary, PLP-NPs represent a promising drug delivery system for the treatment of MDR colon cancer.

## Notes

The details of materials, cell lines and animals, and characterization were presented in the Supporting Information. The methods of acid–base titration method, stability assay, lysosome escape assay, intracellular PTX release evaluation, P-gp expression analysis, ATP content determination, pharmaceutics and biodistribution were also display in Supporting Information. Figures S1–S5 and Table S1 also contained in Supporting Information.

## Supplementary Material

Supplemental Material
