# Supplementary material for: A pH/ROS cascade-responsive and self-accelerating drug release nanosystem for the targeted treatment of multi-drug-resistant colon cancer
Source: Drug Deliv. 2020 Jul 24;27(1):1073–86. doi: 10.1080/10717544.2020.1797238 (PMC7470062; doi:10.1080/10717544.2020.1797238)
Supplement: Supplemental Material [file IDRD_A_1797238_SM1495.docx]

**A pH/ROS cascade-responsive and self-accelerating drug release nanosystem for the targeted treatment of multi-drug-resistant colon cancer**

Chang Na,^#^ Yufei Zhao,^#^ Ning Ge, Liting Qian

Department of Radiation Oncology, Anhui Provincial Cancer Hospital (West District, First Affiliated Hospital of University of Science and Technology of China), Hefei 230000, China

**Corresponding author:**

Liting Qian

**Email:** [money2004@sina.com](mailto:money2004@sina.com)

Department of Radiation Oncology, Anhui Provincial Cancer Hospital (West District, First Affiliated Hospital of University of Science and Technology of China), Hefei 230000, China

**#: These authors contributed equally to this manuscript.**

**Electronic supplementary information**

**Materials**

Paclitaxel (PTX) was purchased from Dalian Meilun Biology Technology Co., Ltd (Dalian, China). Poly histidine (PHis), consisting of twelve histidines, was acquired from Nanjing Leon Biological Technology Co., Ltd (Nanjing, China). Dextran (DEX, molecular weight [MW]: 80 kDa), β-lapachone (β-Lap), 3-mercaptopropionic acid, acetone, 1-Ethyl-3-(3-dimethylaminopropyl) carbodiimide hydrochloride (EDC·HCl), 4-dimethylaminopyridine (DMAP), and triethanolamine (TEA) were purchased from J&K Scientific Ltd (Beijing, China). Dimethyl sulfoxide (DMSO) was supplied by Aladdin Industrial. RPMI-1640 medium and fetal bovine serum were obtained from Invitrogen (Burlington, ON, Canada). Dialysis bags were purchased from Shanghai Greenbird Technology Development Co., Ltd (Shanghai, China). Antibodies, including mouse anti-P-gp and anti-β-actin, were obtained from Abcam Co., Ltd (Cambridge, MA, USA). Secondary antibody (horseradish peroxidase-conjugated goat anti-mouse IgG) was purchased from Boster Biological Technology Co., Ltd (Wuhan, China). 3-(4,5-Dimethyl-2-thiazolyl)-2,5-diphenyl-2-H-tetrazolium bromide (MTT) and phosphate-buffered saline (PBS) were purchased from Beyotime Biotech Co., Ltd (Shanghai, China). Water was purified and deionized using a Milli-Q system acquired from Millipore (Bedford, MA, USA).

**Cell lines and animals**

Human colon cancer cell lines (HCT-8 and HCT-8/PTX cells) and normal NIH3T3 cells were purchased from KeyGEN BioTECH (Nanjing, China). Cells were cultured in RPMI 1640 medium supplemented with 10% fetal bovine serum (FBS) and 100 IU/mL penicillin and 100 µg/mL streptomycin at 37 °C in a humidified atmosphere with 5% CO_2_. The drug resistance of HCT-8/PTX cells was maintained by adding PTX (5 µg/mL) into the medium and culturing in drug-free culture medium for 2 weeks before experiments. NIH3T3 cells were cultured at 37 °C under 5% CO_2_ in DMEM supplemented with 10% bovine calf serum, 1% glutamax, 1% non-essential amino acids, 1% sodium pyruvate, penicillin (50 U/mL), and streptomycin (50 U/mL).

BALB/c nude mice (male, 5–6 weeks old, 18–22 g) and Sprague–Dawley (SD) rats (male, 4–5 weeks old, 45–50 g) were purchased from Charles River (Beijing, China). All animal experiments were performed in accordance with the Guidelines for the Care and Use of Laboratory Animals of University of Science and Technology of China (Anhui, China) and approved by the Animal Ethics Committee of University of Science and Technology of China.

**Characterization**

^1^H NMR spectra were recorded on a Varian U500 (300 MHz) spectrometer. Particle size and polydispersity (PDI) were determined by dynamic light scattering (DLS, ZetaPlus, USA). Cell morphology was observed by transmission electron microscopy (TEM, JEM, Japan). The RP-HPLC protocol used for the analysis of Lapa featured a mobile phase consisting of methanol/H_2_O (9:1, v/v), a flow rate of 1 mL/min, and a UV–vis detection wavelength of 260 nm. The HPLC conditions for PTX analysis featured a mobile phase consisting of methanol/H_2_O (6.5:3.5, v/v), a flow rate of 1 mL/min, and a detection wavelength of 227 nm.

**Acid-base titration**

Acid-base titration was used to evaluate the buffering capacity of PHis.^[1]^ Briefly, PHis (10 mg) was first dissolved in 10 mL solution of NaOH (0.01 M). The pH of this PHis solution was adjusted to 12 using NaOH (1 M). Next, we added HCl (0.01 M) in 50 µL increments. After each addition, the solution was stirred for 5 min, and the pH of the solution measured with a pH meter. The pH value of the PHis solution, along with the volume of HCl added, was then used to plot a titration curve. Endosomal buffering capacity was calculated as the percentage of amine groups protonated from pH 7.4 to pH 5.0 according to equation (4):

(Equation 4) Buffering capacity (%) = $\frac{\left( \text{V}\text{(pH 7.4)-}\text{V}\text{(pH 5.0)} \right)\text{ × 0.1 M}}{\text{N}}\text{ ×100\%}$

where, V (pH 7.4) and V (pH 5.0) present the volume of HCl added for the pH of the solution to reach 7.4 or 5.0, respectively. *N* represents the total number of moles of protonatable amine groups in 1 mg/mL of each PHis.

**Stability assay**

To evaluate the stability of the PLP-NPs and PP-NPs, these two nanosystems were first dispersed in PBS (pH 7.4) with or without 10% FBS, and then incubated at 37 °C with gentle shaking. The size of both NPs was determined by DLS at different predetermined time points (0, 2, 4, 6, 8, 10, 12, and 24 h).

**Lysosomal escape**

To investigate PHis-mediated lysosomal escape, HCT-8/PTX cells were seeded into a confocal microscopy dish and treated with PCP-NPs for 1, 2, or 4 h (400 ng/mL of coumarin-6). Subsequently, cells were incubated with LysoTracker red at 37 °C for 1 h, fixed with 4% polyoxymethylene, stained with DAPI, and monitored by confocal laser scanning microscopy (CLSM) (Zeiss, LSM 710, Oberkochen, Germany).

**Intracellular PTX release**

NIH-3T3 and HCT-8/PTX cells (4 × 10^5^ cells) were seeded into a 60-mm culture dish and treated with PP-NPs or PLP-NPs with or without dicoumarol (60 μM) at an equivalent PTX concentration of 5 μg/mL for 4, 8, and 12 h. Then, 1 mL of cell lysis buffer (1% TritonX-100) was added and the cells were incubated for 30 min. Subsequently, 1 mL of acetonitrile was added to the cell lysate (500 µL), and the lysate was then ultrasonicated for 10 min to extract the drug. The sonicated lysate was centrifuged at 4000 × *g* at 4 °C for 10 min, the supernatant collected, and the concentration of active PTX was detected by RP-HPLC. All measurements of PTX content were normalized to the protein concentration of the cell lysate, as determined by a BCA assay which was used in accordance with the manufacturer’s protocol.

**P-gp expression assay**

HCT-8 and HCT-8/PTX cells were cultured in 60-mm culture dishes for 24 h and then treated with PTX, Lapa, PP-NPs, or PLP-NPs, with or without dicoumarol, at an equivalent Lapa concentration of 2 μg/mL. After 24 h, the cells were collected and the expression of P-gp was evaluated by western blotting. After harvesting, the cells were first mixed with RIPA lysis buffer containing a protease inhibitor cocktail, and incubated on ice for 40 min. Then, the cell lysate was centrifuged at 10,000 × *g* for 10 min to extract proteins. A BCA protein assay kit was used to determine the total protein content. Subsequently, proteins were analyzed by western blotting as described in a previous report.^[2]^

**Determination of ATP content**

The CellTiter-Glo®luminescent cell viability assay kit (Promega, Inc., Madison, WI, USA) was used to determine the cellular ATP content. Briefly, HCT-8/PTX cells were cultured with Lapa, PTX, PP-NPs, or PLP-NPs, with or without dicoumarol, for 4 h. The doses of Lapa and PTX were fixed at 10 µg/mL and 2 µg/mL, respectively. Then, CellTiter-Glo reagent (100 μL) and fresh RPMI 1640 medium (100 μL) were added into each well followed by shaking for 2 min before further incubation at room temperature for 10 min. Subsequently, the solution was analyzed by a microplate reader (Biotek, America). Untreated cells were used as control.

**Pharmaceutics and biodistribution**

SD rats were used as an animal model for all pharmaceutical-related experiments. Rats were randomly divided into two groups (*n* = 5 rats per group) and intravenously administered PTX and PLP-NPs at a dose of 5 mg/kg PTX. At predetermined time points (0, 0.5, 1, 2, 4, 8, 12, and 24 h), blood samples were collected from the orbital venous plexus into heparinized 1.5 mL tubes, and then centrifuged to obtain plasma. Next, acetonitrile was added to the plasma samples, followed by centrifugation (10,000 × *g*, 4 °C, 15 min) to allow collection of the supernatant. Subsequently, the collected samples were dried, redissolved, incubated with H_2_O_2_ (100 mM), and analyzed by RP-HPLC to determine the amount of PTX present. The pharmacokinetic profile of each sample was generated using a two-compartment model.^[3]^

For analysis of biodistribution, we established an HCT-8/PTX tumor-bearing xenograft mouse model by subcutaneously injecting HCT-8/PTX cells (6 × 10^6^) into the right groin of each mouse. When tumor volume had reached approximately 80 mm^3^, the mice were given an intravenous injection of PTX and PLP-NPs at a dose of 5 mg/kg PTX. Then, 24 h post-injection, the mice were sacrificed and the major tissues were excised (spleen, lung, heart, kidney, liver, and tumor). The collected tissues were washed, weighed, and homogenized in 500 μL of acetonitrile, followed by centrifugation (10,000 × *g*, 4 °C, 15 min) to acquire the drug-containing supernatants. Subsequently, the collected samples were dried, re-dissolved, incubated with H_2_O_2_ (100 mM), and analyzed by RP-HPLC to determine the amount of PTX.

**Supporting Figures and Tables**





**Fig S1.** DEX-TK-PTX synthetic routes.


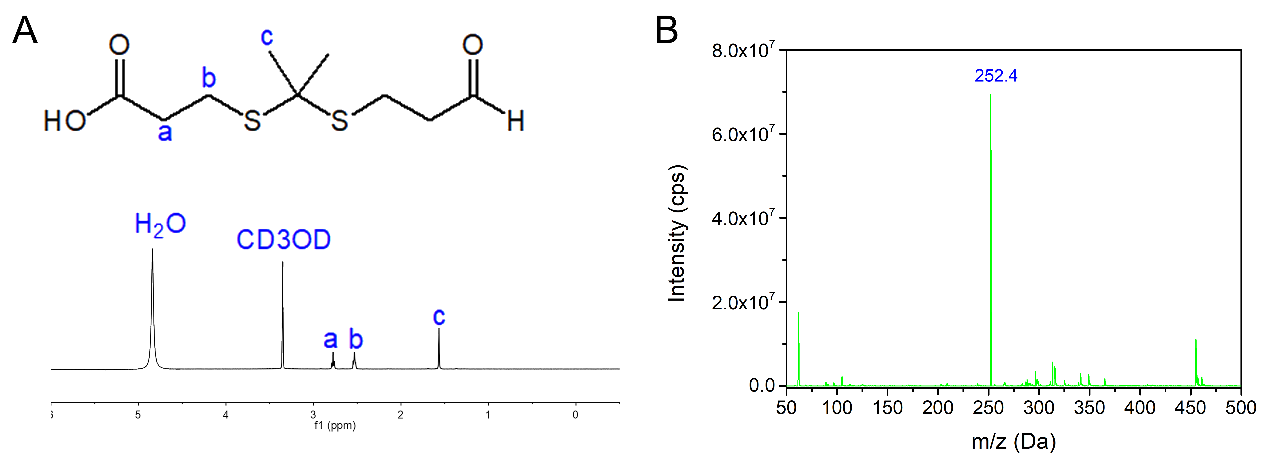


**Fig S2.** Characterization of TK by ^1^H NMR (A) and mass spectrometry (B).


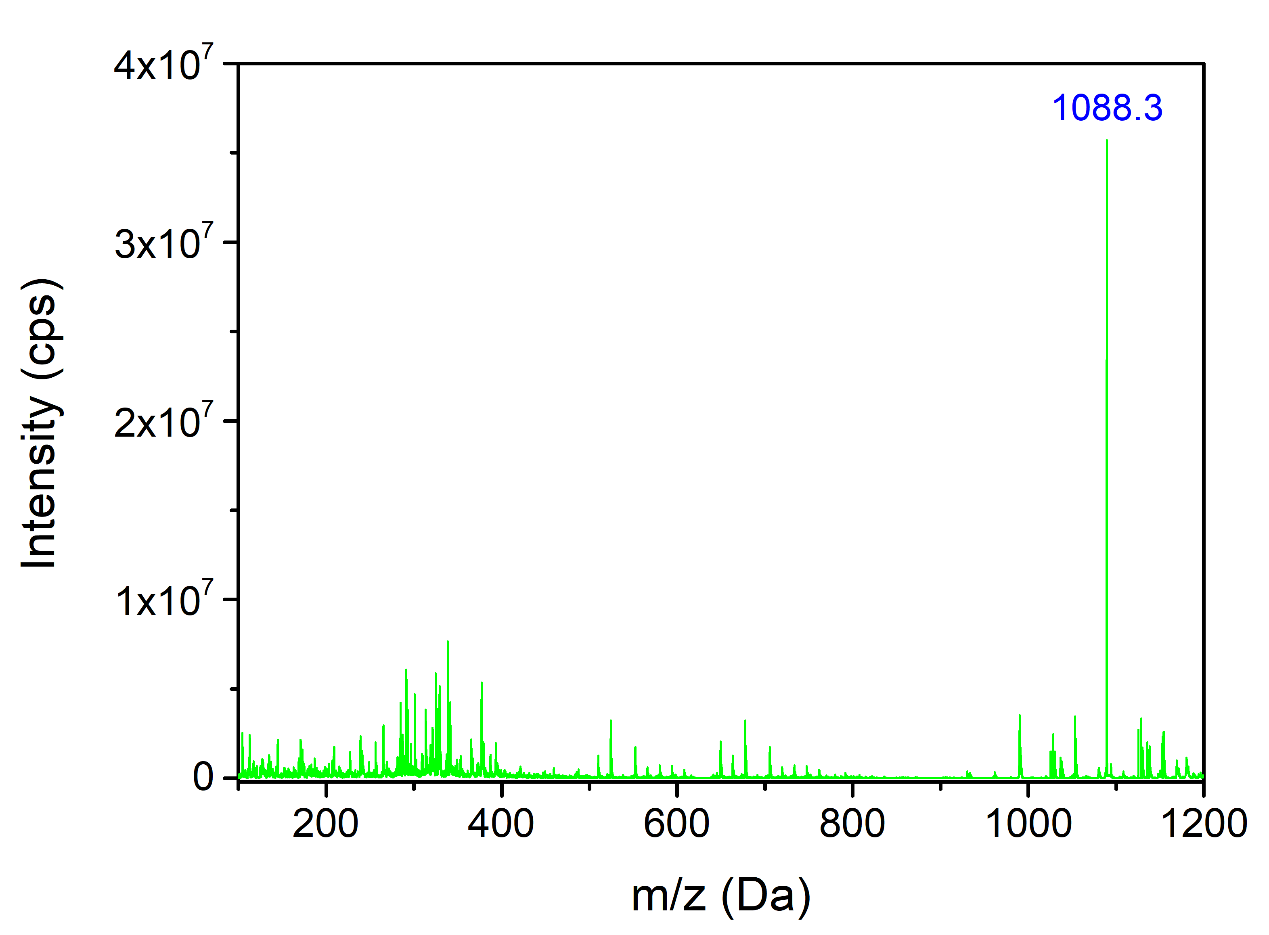


**Fig S3.** Mass spectrometry analysis of TK-PTX.


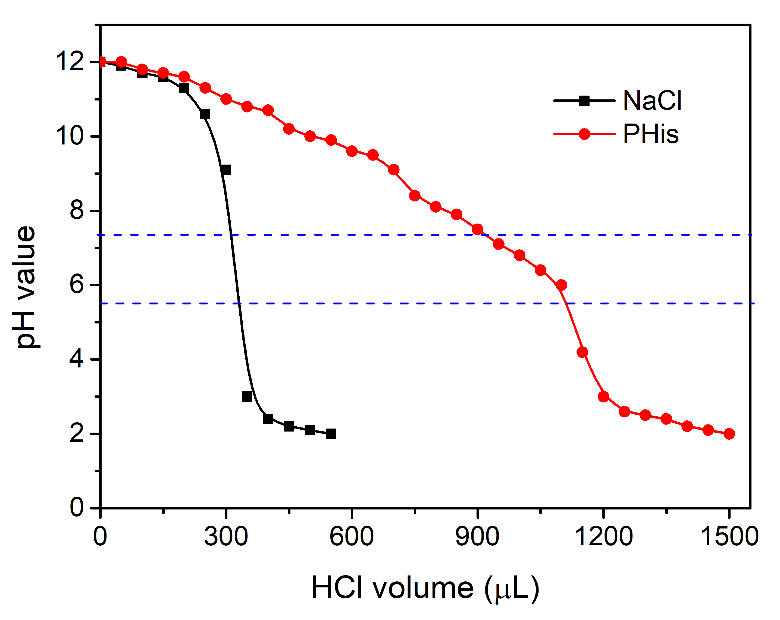


**Fig S4.** pH profile of PLH−PEG and NaCl.


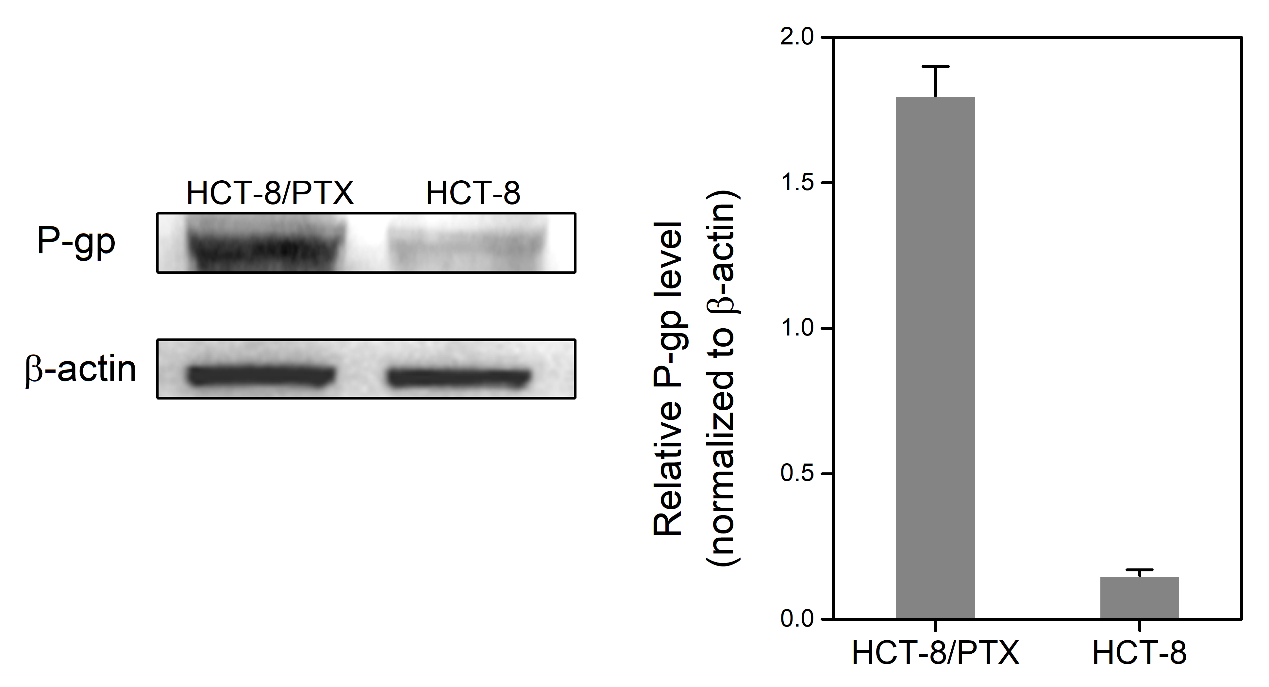


**Fig S5.** Western blotting analysis of P-gp expression in HCT-8 and HCT-8/PTX cells (*n* = 6).

| **Table S1**  Characterization of DEX-TK-PTX. | | | | |
| --- | --- | --- | --- | --- |
| Prodrug | DBE (wt.%) | DBC (Wt.%) | DBR (%) | CMC (μg/mL) |
| DEX-TK-PTX-1 | 76.5 | 1.5 | 0.9 | - |
| DEX-TK-PTX-2 | 74.3 | 5.3 | 3.1 | - |
| DEX-TK-PTX-3 | 71.2 | 10.2 | 6.3 | 89.2 |
| DEX-TK-PTX-4 | 73.8 | 15.4 | 8.7 | 19.8 |
| DEX-TK-PTX-5 | 74.2 | 20.2 | 12.8 | 4.3 |
| DEX-TK-PTX-6 | 74.3 | 27.2 | 16.3 | 59.6 |
| DEX-TK-PTX7 | 72.9 | 38.5 | 23.1 | 234.7 |

**References**

[1] Zhang X, Chen D, Ba S, et al. Poly(l-histidine) based triblock copolymers: pH induced reassembly of copolymer micelles and mechanism underlying endolysosomal escape for intracellular delivery. *Biomacromolecules* 2014;15:4032-45.

[2] Tomono T, Yano K, Ogihara T. Snail-induced epithelial-to-mesenchymal transition enhances P-gp-mediated multidrug resistance in HCC827 Cells. *Journal of pharmaceutical sciences* 2017;106:2642-9.

[3] Zhang C, Qu G, Sun Y, et al. Pharmacokinetics, biodistribution, efficacy and safety of N-octyl-O-sulfate chitosan micelles loaded with paclitaxel. *Biomaterials* 2008;29:1233-41.
